# BABA-Induced DNA Methylome Adjustment to Intergenerational Defense Priming in Potato to *Phytophthora infestans*

**DOI:** 10.3389/fpls.2019.00650

**Published:** 2019-05-31

**Authors:** Daniel Kuźnicki, Barbara Meller, Magdalena Arasimowicz-Jelonek, Agnieszka Braszewska-Zalewska, Andżelika Drozda, Jolanta Floryszak-Wieczorek

**Affiliations:** ^1^Department of Plant Physiology, Poznań University of Life Sciences, Poznań, Poland; ^2^Department of Plant Ecophysiology, Faculty of Biology, Adam Mickiewicz University in Poznań, Poznań, Poland; ^3^Department of Plant Anatomy and Cytology, Faculty of Biology and Environmental Protection, The University of Silesia in Katowice, Katowice, Poland

**Keywords:** potato acquired resistance, *Phytophthora infestans*, stress-responsive genes, DNA methylation/demethylation, intergenerational resistance

## Abstract

We provide evidence that alterations in DNA methylation patterns contribute to the regulation of stress-responsive gene expression for an intergenerational resistance of β-aminobutyric acid (BABA)-primed potato to *Phytophthora infestans*. Plants exposed to BABA rapidly modified their methylation capacity toward genome-wide DNA hypermethylation. *De novo* induced DNA methylation (5-mC) correlated with the up-regulation of Chromomethylase 3 (CMT3), Domains rearranged methyltransferase 2 (DRM2), and Repressor of silencing 1 (ROS1) genes in potato. BABA transiently activated DNA hypermethylation in the promoter region of the *R3a* resistance gene triggering its downregulation in the absence of the oomycete pathogen. However, in the successive stages of priming, an excessive DNA methylation state changed into demethylation with the active involvement of potato DNA glycosylases. Interestingly, the 5-mC–mediated changes were transmitted into the next generation in the form of intergenerational stress memory. Descendants of the primed potato, which derived from tubers or seeds carrying the less methylated *R3a* promoter, showed a higher transcription of *R3a* that associated with an augmented intergenerational resistance to virulent *P. infestans* when compared to the inoculated progeny of unprimed plants. Furthermore, our study revealed that enhanced transcription of some SA-dependent genes (*NPR1, StWRKY1*, and *PR1*) was not directly linked with DNA methylation changes in the promoter region of these genes, but was a consequence of methylation-dependent alterations in the transcriptional network.

## Introduction

Plants exposed to adverse environmental conditions are forced to develop multiple and diverse strategies to sense the stress and mount defense responses against it. Defense priming is postulated as the physiological state of a plant where adaptive responses, initiated by various stimuli, trigger its metabolism so that the plant responds more effectively when second and more severe stress is encountered (Prime-A-Plant Group et al., 2006). It has been well documented that the non-protein amino acid, namely β-aminobutyric acid (BABA), effectively mediates the conditioning of induced plant defense mechanisms leading to enhanced protection against various pathogens (Cohen et al., 2016). Although BABA pre-treatment or other stimuli may prime a plant against future stress (Floryszak-Wieczorek et al., 2015; Meller et al., 2018), there is also evidence that such stress memory establishment is a relatively rare event or priming is reset during the period of stress recovery (Zimmerli et al., 2000; Pecinka et al., 2009; Crisp et al., 2016; Hilker et al., 2016).

The duration of the priming memory may often be short (days or weeks), but in some cases, it may extend to the first stress-free generation (F_1_), designated as “intergenerational memory,” or to at least two stress-free generations (F_2_), designated as “transgenerational memory” (Lämke and Bäurle, 2017). Recent findings revealed that primed parent could transmit to its offspring an improved response to potential future stress, in a process that is of epigenetic nature (Avramova, 2015; Conrath et al., 2015; Mauch-Mani et al., 2017).

Epigenetic regulation of gene expression relies on DNA methylation, small RNA activity and histone modifying complexes, which regulate chromatin structure. Alterations in DNA methylation have been suggested to be involved in the process of heritable stress adaptation and correlate with changes in stress-responsive gene expression (Zhu et al., 2015; Crisp et al., 2016; Seidl et al., 2016). DNA methylation (5-mC) is a type of epigenetic mark that modifies chromatin structure and gene expression, resulting often (but not always) in compacted chromatin and transcriptional gene silencing (TGS). It is well known that DNA methylation in plants is found in all cytosine sequence contexts (CG, CHG, CHH; where H = A, C or T) and the different types may serve as different functions. DNA methylation is relatively stable and may be maintained during cell division and differentiation. The transposon silencing and prevention of paramutation are essential to ensure cell function. However, under unfavorable conditions, DNA methylation patterns might be prone to changes, which may contribute to the exhibition of new resistance traits, or may give rise to abnormalities in the patterns of 5-mC associated with plant diseases. The genome-wide methylation pattern is dynamically regulated by three independent processes: maintenance of methylation, *de novo* methylation and DNA demethylation (Zhang et al., 2018).

An important component in the activated methyl cycle (AMC) determining the cellular methylation potential under normal and stress conditions is S-adenosyl-L-homocysteine hydrolase (SAHH), which catalyzes the reversible hydrolysis of S-adenosylhomocysteine (SAH) to adenosine and homocysteine (Palmer and Abeles, 1979). Subsequently, homocysteine is then converted, through methionine, to S-adenosylmethionine (SAM) and acts as a methyl donor for methyltransferases (MTs) in *trans*-methylation reactions of various acceptors, i.e., DNA, proteins and lipids. The regeneration of SAM from methionine, driven by SAM-synthase (SAMS), is the last step of the AMC cycle. However, several lines of evidence have indicated that SAHH plays a key role in the maintenance of the methylation potential under physiological or pathological conditions through regulating the inter- and intra-cellular SAH/SAM ratio (Rahikainen et al., 2018).

Plant DNA methyltransferases (DRMs) are grouped into three families: CG methylation is mediated mainly by Methyltransferases (METs), CG and CHG methylation is regulated by Chromodomain methyltransferases or CMTs, respectively, while CHH methylation is mainly catalyzed by Domains Rearranged DRMs. *De novo* DNA methylation is predominantly controlled in *Arabidopsis* by the RNA-directed DNA methylation (RdDM) pathway, in which methylated siRNAs are loaded onto ARGONAUTE4 (AGO4) to form the RNA-induced silencing complex that promotes recruitment of DRM2 to the target locus (Henderson et al., 2010; Saze et al., 2012; Matzke and Mosher, 2014). The current state of DNA methylation patterns under normal or stress circumstances is often the effect of cooperation or competition of these three methyltransferases and the RdDM pathway with the DNA demethylation machinery (Lei et al., 2015).

The active DNA demethylation in *Arabidopsis* is performed by four DNA glycosylases, DEMETER (DME), DME-like (DML2 and DML3) and Repressor of silencing (ROS1), with the latter to be preferentially involved in counteracting *de novo* DNA methylation established by the RdDM pathway (Zhu, 2009; López Sánchez et al., 2016).

Plants under stress have to balance between the stable and flexible DNA methylation status tuned with the transcriptional repressive or active state of stress-responding genes. The concept of memory-type transcription during repeated stresses indicates that sometimes the information on priming is stored in the form of epigenetic marks which contribute to the regulation of subsequent transcription of salicylic acid (SA) dependent stress-related genes (Pecinka et al., 2009; Jaskiewicz et al., 2011; Avramova, 2015). An elevated level of SA, a key component of defense-related signal transduction, induces SA-dependent changes that are mainly controlled by the central immune regulator Non-expressor of Pathogenesis-Related Genes 1 (NPR1). The NPR1 as a transcription coactivator, upon interaction with transcription factors TGA and WRKY (recognizing the W-box regulatory element), is required for SA-dependent regulation of *Pathogenesis-Related* (*PR*) gene expression and the establishment of the priming state (Fu and Dong, 2013). The *StWRKY1* transcription factor is associated with potato immunity to *Phytophthora infestans*, the causal agent of late blight disease of potato, and the *StWRKY1*-silenced potato leaves exhibited an increase in the pathogen biomass and correlated with a lower level of late blight resistance (Yogendra et al., 2015).

In turn, genome instability connected with the altered DNA methylation status in the promoter regions of respective resistance (*R*) gene clusters could contribute to phenotypic plasticity in response to pathogens promoting the formation of new patterns of resistance genes regulation (Alvarez et al., 2010; Spoel and Dong, 2012). The group of the *R* genes coding for the nucleotide-binding site, leucine-rich repeat (NBS-LRR) proteins, that recognize pathogen effectors, is one of the largest families found to be regulated by micro RNAs (miRNAs) that trigger secondary siRNA production (Richard et al., 2018). At least 11 race-specific late blight *R* genes, deriving from *Solanum demissum* have been incorporated into various potato cultivars, belong to the family of NBS-LRR encoding resistance genes (Vleeshouwers et al., 2011). The *R3a* resistance gene has been widely introduced into potato cultivars and it is essential for plant immunity by encoding *R3a* protein recognizing the *P. infestans* effector Avr3a (Bos et al., 2010).

It was generally accepted that host plant harboring specific *R* genes encode proteins recognizing the corresponding pathogen-produced effector proteins (avirulence factors or Avr) and as a result inducing effector-triggered immunity (ETI) in the form of a strong defense response. In turn, the recognition of the host plant conserved pathogen-associated molecular patterns (PAMPs) results in PAMP-triggered immunity (PTI), which elicits a “general defense response” or basal immunity. Because pathogens have evolved a broad range of effectors to dampen PAMP-triggered signals and attenuate PTI, this type of defense response is much weaker than ETI (Pedley and Martin, 2003; Jones and Dangl, 2006).

The priming stimulus or pathogen ingress typically involves host genome hyper- or hypomethylation, which can influence the biogenesis of small RNAs, together with the expression of the *R* genes (Alvarez et al., 2010; Weiberg and Jin, 2015). However, to date, no model has been proposed for the DNA methylation state associated with its silencing or activating role in the expression of stress-responsive genes. In rice, chemically induced hypomethylation revealed suppression of *R* gene silencing (*Xa21G*) concomitant with enhanced and heritable resistance to *Xanthomonas oryzae* pv. *oryzae* (Akimoto et al., 2007). Reduction in the DNA methylation level may affect the stability of the *R* genes belonging to the NBS-LRR defense gene family; members of the family are clustered in chromosome regions abundant in repetitive sequences and transposon elements (TEs), rich in repressive chromatin modifications, such as DNA and H3K9me3 methylation (Saze et al., 2012; Du et al., 2015). Together with small RNAs, these epigenetic modifications preserve transcriptionally silent chromatin and prevent TEs expression. Transcription of the *R* genes needs to be strictly controlled to avoid inappropriate activation, since redundant *R* gene expression, in the absence of the pathogen, could affect plant growth and development (Xia et al., 2013). In turn, after pathogen recognition some 5-mC marks may be erased from these regions that can promote TEs activation, while the *R* genes (although not all) are moderately upregulated, presumably for pathogen recognition, amplify immune signals and enhance or reset resistance against the aggressor (Navarro et al., 2004).

The global genome methylation level significantly varies between species, ranging from 6–14% of total cytosines in *Arabidopsis*, 20% in wheat, 25% in maize, to more than 35% in potato (Kakutani et al., 1999; Steward et al., 2002; Xin et al., 2015; Viggiano and de Pinto, 2017). Surprisingly, the few organisms lacking an efficient 5-mC DNA methylation system include *Drosophila* (<0.0002%), *Caenorhabditis elegans*, yeast and oomycetes (Capuano et al., 2014). *P. infestans* is a member of the oomycetes, a group of organisms phylogenetically separated from fungi and grouped with the golden-brown algae in the stramenopiles (Burki et al., 2007). In this study, the changes in the DNA methylation status of potato in its interaction with *P. infestans* were examined.

Recently we found that the offspring of BABA-primed potato cv. Sarpo Mira maintained a higher resistance to virulent *P. infestans* MP977 over one BABA-untreated generation. The H3K4me2 epigenetic mark was identified in high numbers on the gene body of *StWRKY1, PR1*, and *PR2* concomitant with transcript downregulation detected in leaves of primed potato and its descendants before the triggering stress. Moreover, our previous study revealed that the primed state in potato was associated with the transcriptional memory to post-infection histone acetyltransferase (HAT) activation in the next generation after the challenge inoculation with *P. infestans* (Meller et al., 2018). In the present study, with the same experimental design as mentioned above, we investigated how DNA methylation and demethylation in potato contribute to BABA-primed changes associated with transcriptional memory to improve resistance against the virulent *P. infestans* MP977.

## Materials and Methods

### Plant Material and BABA Treatment

Plants of the tetraploid potato cv. Sarpo Mira came from the Potato Genebank (Plant Breeding and Acclimatization Institute – IHAR-PIB in Bonin, Poland). Potato cultivar Sarpo Mira (carrying the *R* genes: *R3a, R3b, R4, Rpi-Smira1*, and *Rpi-Smira2*) is highly resistant to avr *P. infestans*. Potato seedlings, propagated through *in vitro* culture, were transferred to the soil and were grown to the stage of ten leaves in a phytochamber with 16 h of light (180 μmol⋅m^-2^⋅s^-1^) at 18 ± 2°C and 60% humidity. Then the plants were immunized by spraying leaves with 5 mM of BABA (5 ml per plant). The non-immunized plants were sprayed with water. Three days after BABA treatment (at 72 h) potato plants were challenge inoculated with virulent (vr) *P. infestans* (isolate MP977). Plants were kept for 24 h at 100% humidity and 18°C to facilitate disease development and then transferred to normal growth conditions. Leaves were collected at 1, 3, 6, 24 and 48 h after *P. infestans* challenge inoculation and frozen in liquid nitrogen until analysis.

The F_1_ plants, i.e., vegetative (from tubers) and generative (from seeds) progeny, were obtained from non-primed and BABA-primed selfed parental plants (F_0_). According to the experimental design (Supplementary Figure S1), the parental line (F_0_) was treated once with 5 mM BABA and the progeny (F_1_) was only inoculated with *P. infestans* MP977 at the 10 compound leaf stage.

### Pathogen Culture and Plant Inoculation

The vr *P. infestans* (Mont.) de Bary isolate MP977 (A1 mating type, race 1.2.3.4.6.7.10) and the avirulent *P. infestans* isolate MP946 (A1 mating type, race 1.3.4.7.10.11) were kindly provided by the Plant Breeding Acclimatization Institute (IHAR), Młochów Research Centre, Poland. The MP946 avirulent isolate of *P. infestans* encoding AvrR3a in the presence of the corresponding *R3a* gene product of cv. Sarpo Mira elicits ETI, a strong response associated with the hypersensitive reaction (HR) in the host plant, which prevents pathogen colonization. In contrast, the virulent isolate MP977 of *P. infestans* triggers PTI, resulting in late blight symptom development in cv. Sarpo Mira. The pathogen was grown on a rye medium (Caten and Jinks, 1968) and transferred through a potato tuber two times before infection. Sporangia of *P. infestans* were obtained by washing 14-day-old cultures with cold distilled water. Zoospores were released by incubating the sporangia in water at 4°C for 2 h. Potato plants were inoculated by spraying 3 ml of a freshly isolated suspension of sporangia and zoospores with approximately 2.5 × 10^5^ sporangia per ml. The mock-inoculated plants were sprayed with water.

### Quantification of *P. infestans*

The *P. infestans translation elongation factor 1a* (*Pitef1*) gene, a single copy constitutively expressed gene, was used to quantify *P. infestans* in inoculated potato leaves using RT-qPCR analysis. For all the samples, the transcript levels of the *Pitef1* gene were calculated relative to the expression levels of the potato *ef1α* and *18S rRNA* (van’t Klooster et al., 2000; Orłowska et al., 2012).

### Assessment of Disease Development

Disease symptoms were determined at day 5 after inoculation (dpi), based on the estimation of trypan-blue stained leaves (Wilson and Coffey, 1980) inoculated with zoospore suspension. Imaging was performed by scanning leaf discs with a LIDE 210 Scanner (Canon). The blue stain corresponded to the area covered by *P. infestans* mycelium and was analyzed using the ImageJ 1.47v open source software (Wayne Rasband National Institutes of Health, United States).

### Global 5-mC Detection

Global 5-mC detection was performed with the 5-mC ELISA kit (Zymo Research, United States) according to the manufacturer’s protocol. All DNA samples were isolated from 200 mg of frozen leaf tissue using the Wizard^®^ Genomic DNA Purification Kit (Promega, United States) following the manufacturer’s protocol.

### Imaging of DNA Methylation

The immunostaining procedure was carried out as described by Braszewska-Zalewska et al. (2014). All probes were fixed in 3:1 methanol/glacial acetic acid to detect DNA methylation, subsequently macerated in 20% pectinase (Sigma Aldrich) and 2% cellulase (Onozuka) in 0.01 mM sodium citrate buffer (pH 4.8). After digestion, the material was washed again with sodium citrate buffer. Then the vascular bundles and the epidermis were removed. Cell suspensions were transferred to fresh 1.5 ml tubes and centrifuged (10000× *g*). The supernatant was removed and cells were washed with 45% acetic acid, centrifuged and suspended in acetic acid and heated up to 65°C for a minute. Next, the cells were transferred to a glass slide, covered with a coverslip and frozen in liquid nitrogen for 30 s. After freezing and removal of the coverslips, the slides were dried. Global cytosine methylation was immunodetected with mouse antibodies raised against 5-mC (Abcam; 1:200 in 1% BSA in 1 × PBS). Samples were denatured in 70% formamide in 2 × SSC for 2 min at 70°C. Then samples were dehydrated in 70 and 100% ethyl alcohol for 5 min and air-dried. Next, probes were blocked with 5% BSA and incubated with the primary antibody at 37°C for 1 h and afterward, they were washed in PBS and incubated with the secondary antibody (Alexa Fluor^488^ goat anti-mouse IgG; Invitrogen, Molecular Probes) applied under the same conditions. Chromosomes were counterstained with 2.5 mg/ml 4′,6-diamino-2-phenylindole (DAPI, Sigma Aldrich) in the Vectashield (Vector Laboratories). The quantitative acquisition and analysis were performed using a high-content screening system (ScanˆR, Olympus) based on a wide-field Olympus IX81 microscope, equipped with a CCD ORCA-ER camera (Hamamatsu Photonics, Japan) and an MT20 illumination system based on a 150-W xenon-mercury lamp. Automated segmentation of nuclei was based on threshold values (a border value of the fluorescence intensity of pixels between the background and the object). ImageJ (ver.1.52a ^1^) was performed assuming the following parameters of fluorescence intensities: “total” (the sum of pixel intensity divided by the area of the object) and “mean” (total intensity divided by the area of the object). Normality of the signal intensity was assessed for each analyzed group (the Chi-square goodness-of-fit test, *p*<0.05). As all the samples were large (*N* = 780 to 3,870), the *t*-test for independent statistical samples was used to verify significant differences between the control and the treated samples.

### Gene Expression Analysis

Gene expression analysis was performed for *SAMS* and *SAHH* that constitute important elements of the plant activated cellular methyl cycle (AMC). Maintenance of the DNA methylation/demethylation status of the potato genome was verified by transcript abundance analyses for the following DRMs *MET1, CMT3, DRM2* and demethylases *StDML2* (ortholog to *Arabidopsis* At3g47830) and *ROS1*. Moreover, RT-qPCR analysis was carried out for the key potato resistance *R3a* gene and SA-dependent *NPR1, StWRKY1, PR1* stress-responding genes. RNA from 150 mg of frozen leaf tissue was extracted using the TRI Reagent^®^ (Sigma, United States). DNA was removed using a Deoxyribonuclease Kit (Sigma, United States) and reverse transcription of 1 μg RNA was performed using a Reverse Transcription Kit (Thermo Fisher Scientific, United States). The RT-qPCR assays were performed applying the following conditions: 10 min at 95°C, followed by 35 cycles of 10 s at 95°C, 5 s at the annealing temperature of each specific primer (Supplementary Table S1) and 10 s at 72°C. The reaction mixture contained 0.1 μM of each primer, 1 μl of 5 × diluted cDNA, 10 μl of the Power SYBR Green PCR Master mix (Applied Biosystems, United States) and DEPC treated water to the total volume of 20 μl. The PicoReal Thermocycler (Thermo Fisher Scientific, United States) was used for RT-qPCR analyses. Primers were designed based on the available databases of NCBI (National Center of Biotechnology Information, United States) or PGSC (Potato Genome Sequencing Consortium) using the Primer-Blast software and are listed in Supplementary Table S1. The data were normalized to the reference genes, namely the elongation factor *ef1α* (AB061263) and the *18S rRNA* (X67238). The Ct values were determined with the use of a Real-time PCR Miner (Zhao and Fernald, 2005) and the relative gene expression was calculated with the use of the efficiency corrected calculation models proposed by Pfaffl (2001) and Tichopad et al. (2004).

### Evaluation of Specific DNA Methylation Levels Using Methylation-Sensitive High Resolution Melting (MS-HRM) Analysis

High-Resolution Melting (HRM) PCR is a commonly used method in genotyping or single nucleotide polymorphism detection (Simko, 2016). In combination with bisulfite treatment (methylation sensitive HRM, MS-HRM), where conversion of non-methylated cytosine to uracil causes changes in the DNA melting curve, HRM technique may be applied to detect qualitative changes in DNA methylation of targeted sequences both in human cancer diagnostic research (Wojdacz et al., 2008) and in plants (Tricker et al., 2012). Potato DNA (1 μg) was treated with sodium bisulfite using the EZ DNA Methylation-Gold Kit (Zymo Research, United States). Primers specific to bisulfite-converted antisense DNA were selected to amplify a region of a CpG island in the promoter regions for the *NPR1, PR1, R3a* and *StWRKY1* genes using the MethPrimer 2.0 software. The gene sequences were obtained from the NCBI database (Supplementary Table S2). MS-HRM was performed in 20 μl reaction mixtures containing 10 μl of 2 × Luminaris Color HRM Master Mix, 200 ng of bisulfite-treated DNA, and 0.5 μM of each primer. MS-HRM was initiated by incubation for 5 min at 95°C to denature the DNA, followed by 50 cycles of 5 s at 95°C, 10 s at the annealing temperature of each specific primer and 15 s at 72°C. Fluorescence was measured on a PicoReal Thermocycler once per cycle to monitor template melting during a linear temperature transition from 65 to 95°C at 0.1°C/s. Fluorescence data were converted into melting peaks by the PicoReal software (ver. 2.2) to plot the negative derivative of fluorescence over temperature vs. temperature (-dF/dT vs. T) (Supplementary Table S1). The control was selected as a reference curve, while the difference between each curve and the reference was plotted against temperature to give a “difference curve” plot (Wittwer et al., 2003). The original reference curve became a horizontal line at zero, while the differentially methylated genotypes clustered along different paths to facilitate visual discrimination with the positive/negative values on the *Y*-axis for mean hypermethylation/hypomethylation, respectively. From the obtained data mean and standard deviations were calculated for each temperature point.

### Statistical Analysis

All the results were based on three independent experiments, each with at least three biological replicates. For each experiment, means of the obtained values were calculated along with standard deviations. The analysis of variance was conducted and the least significant differences (LSDs) between means were determined using Tukey’s test at the level of significance α = 0.05 and 0.01.

## Results

### BABA-Primed Protection Against Late Blight Disease

**FIGURE 1:**
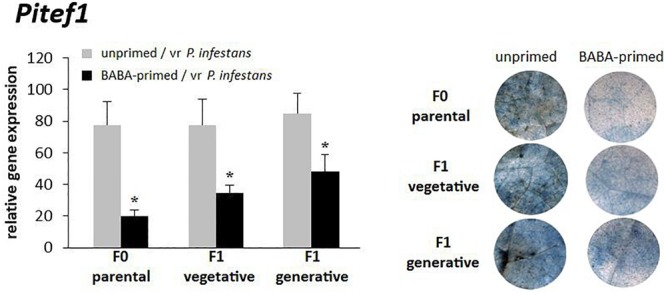
Intergenerational resistance of potato against *P. infestans*. BABA-induced resistance against *P. infestans* in the parental line (F_0_); intergenerational resistance in the vegetative F_1_ progeny and the generative F_1_ progeny. Three days after BABA treatment potato leaves were challenged with *P. infestans* and *Pitef1* gene expression at 48 hpi and trypan-blue stained leaves at day 5 (dpi) were analyzed. Values represent means of at least three independent experiments, each with at least three biological replicates. Asterisks (^∗^) indicate values that differ significantly from unprimed and vr *P. infestans*-inoculated potato leaves at α<0.05, respectively.

The durability of BABA-primed resistant responses in potato cv. Sarpo Mira was evaluated on the basis of pathogen gene expression in the inoculated parental line and the vegetative progeny of primed plants derived from tubers and progeny derived from seeds. The BABA-priming efficiency as expressed by the transcript level of *Pitef1* and cytochemical assay also corresponded to the reduced level of leaf colonization by *P. infestans*, as described previously (Meller et al., 2018). BABA treatment, prior to the challenge inoculation, resulted in a more pronounced resistance against late blight disease in potato (F_0_), since a 4-fold lower level of *Pitef1* transcription at 48 hpi was observed when compared to unprimed inoculated plants (Figure 1). Descendants of BABA-primed potato (F_1_), grown from tubers, showed approx. 2.5-fold reduction in *Pitef1* transcript accumulation at 48 hpi. Intergenerational resistance against *P. infestans* MP977 was also found in the progeny obtained from seeds (F_1_). The protection of this generation was reflected in the *Pitef1* transcript being approx. 2-fold lower at 48 hpi than in unprimed inoculated plants. The less of pathogen biomass in the F_0_ and F_1_ lines of BABA-primed plants positively correlated with reduced disease symptoms detected by trypan blue staining of *P. infestans* mycelium (Figure 1). Our results revealed that potato exposed to BABA displayed enhanced resistance to *P. infestans* and produced offspring that were more resistant against the late blight disease than those from unprimed plants regardless of the means of reproduction.

### BABA Treatment Is Effective in Modifying the Cellular Methylation Potential

To examine whether the primed state providing better stress resistance in potato and its progeny, was due to the modification of the DNA methylation status, we first examined how BABA might alter gene expression of *SAMS* and *SAHH*, both being important components of the plant activated cellular methyl cycle.

**FIGURE 2:**
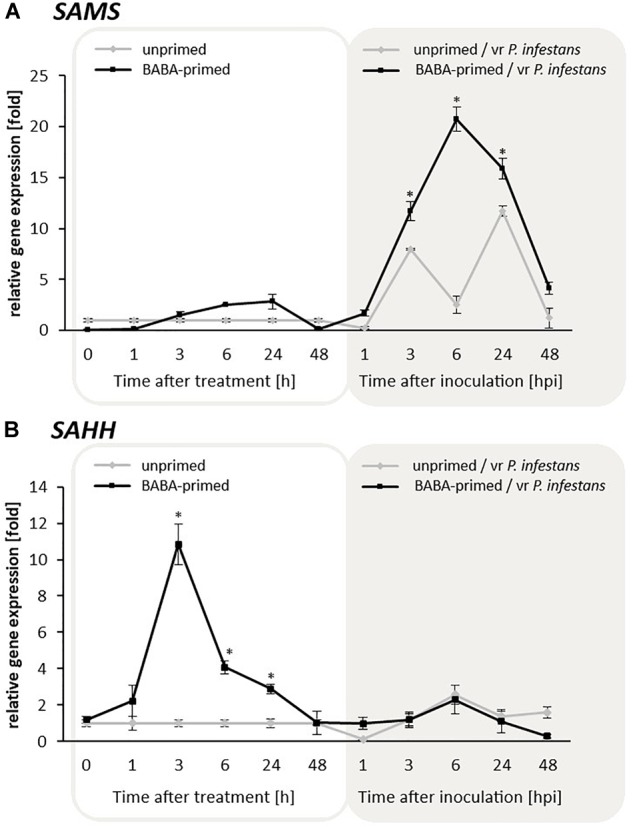
Expression patterns of *SAMS* **(A)** and *SAHH* **(B)** contributing to the potato cellular methylation potential in response to BABA treatment and challenge inoculation with *P. infestans* MP977 (3 days after BABA treatment). Analyses were performed at 1–48 h after 5 mM BABA exposure (white background) and 1–48 hpi after challenge inoculation with vr *P. infestans* (gray background). Light lines refer to unprimed and dark lines to primed plants. Values represent mean ± SD of at least three independent experiments. Asterisks (^∗^) indicate values that differ significantly from unprimed (water treated) or unprimed and *P. infestans* inoculated potato leaves at α<0.05, respectively.

The obtained data clearly indicated that BABA at a concentration of 5 mM enhanced *SAHH* transcription level starting from 1 to 24 h and slightly intensified *SAMS* expression at the same time after BABA supplied (Figure 2A,B). In turn, primed potato exposed to *P. infestans* MP977 inoculation showed abundant transcript accumulation mainly for *SAMS* (peaking at 6 hpi after pathogen attack) (Figure 2A,B). It cannot be excluded that the increase of *SAMS* expression after inoculation was a result of higher expression of *SAHH* and a consequently larger production of *SAM* precursors that had to be processed. Moreover, our experiment revealed that BABA-primed rather indirect epigenetic effect, through modulation of the SAHH/SAM ratio, caused instability in the cellular methylation potential with rather an opposite trend in changes upon challenge inoculation.

### DNA Methylation/Demethylation Genes Are Involved in the BABA-Primed Defense

**FIGURE 3:**
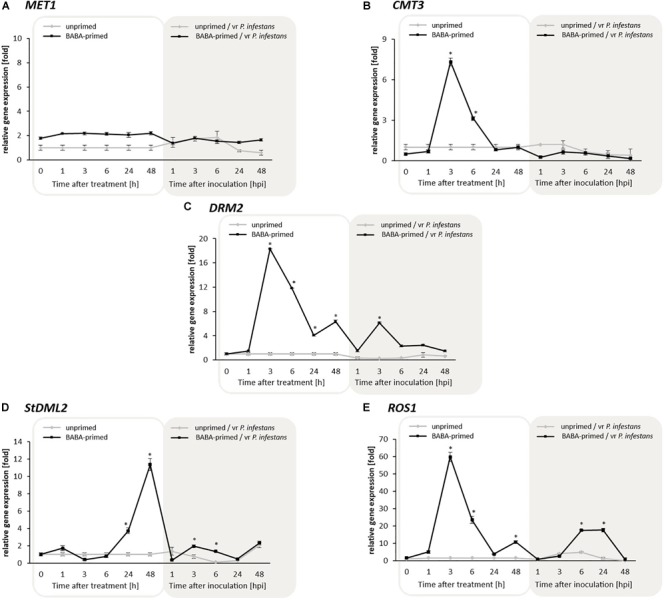
Transcriptional changes in potato DNA methylating genes *MET1* **(A)**, *CMT3* **(B)**, *DRM2* **(C)**; and DNA demethylating genes *StDML2* **(D)**, and *ROS1* **(E)** in response to BABA and challenge inoculation with vr *P. infestans* MP977 (3 days after BABA treatment). Analyses were performed at 1–48 h after 5 mM BABA treatment (white background) and 1–48 hpi after challenge inoculation with *P. infestans* (gray background). Light lines and dark lines refer to unprimed and primed plants, respectively. Values represent mean ± SD of at least three independent experiments. Asterisks (^∗^) indicate values that differ significantly from unprimed (water-treated) or unprimed and *P. infestans* inoculated potato leaves at α<0.05 (^∗^), respectively.

Since DNA methylome plasticity is due to the presence of various enzymes modifying DNA methylation patterns, we first analyzed DRMs to investigate a potential association between cytosine methylation and transcription upon BABA-priming. RT-qPCR assays of *MET1*, required for global cytosine methylation maintenance in plants, did not show significant changes in its expression either after the BABA treatment or challenge inoculation. Thus, *MET1* activity did not seem to be important for DNA reprogramming methylation induced by BABA (Figure 3A).

In turn, an enhanced *CMT3* gene expression (7-fold increase) was found at 3 h upon BABA treatment (Figure 3B). Interestingly, the 18-fold increase in *DRM2* transcription was recorded at the same time after inducer exposure (Figure 3C). This data indicated the essential role and involvement of *CMT3* and *DRM2* in *de novo* DNA methylation associated with BABA priming.

Then we focused on the role of active DNA demethylation in BABA priming, driven by DNA glycosylase, namely DEMETER-like DNA demethylase (At3g47830), referred to as *StDML2*, and the *Repressor of silencing 1* (*ROS1*) antagonists of RdDM to prevent DNA hypermethylation at specific loci. *ROS1* underwent a strong 60- and 20-fold transcriptional increase at 3 and 6 h after BABA treatment, respectively; in the following hours the expression rapidly diminished (Figure 3E). Interestingly, *ROS1* transcript level showed time-dependent kinetics similar to that of *DRM2* expression upon BABA treatment and challenge inoculation with vr *P. infestans*, which confirmed their functional cooperation both controlled by RdDM. At the later phase of priming (from 24 to 48 h) an overproduction (12-fold increase) of *StDML2* transcript was observed, suggesting active demethylation of the potato DNA methylome (Figure 3D). Obtained results revealed that BABA-induced wave of DNA methylation followed by removal of this methylation mark might change the target genomic region of key importance for long-lasting priming for defense.

### Primed Potato Plants Exhibit a Shift in Global DNA Methylation Levels

To evaluate whether the genome-wide methylation status of potato was modified by BABA we performed global genome-methylation analyses using immunocytochemical (ICC) and ELISA immunoassay, as well as MS-HRM analysis in order to determine methylation patterns of the promoter region in the pathogen-responsive genes.

### Global DNA Methylation Changes

**FIGURE 4:**
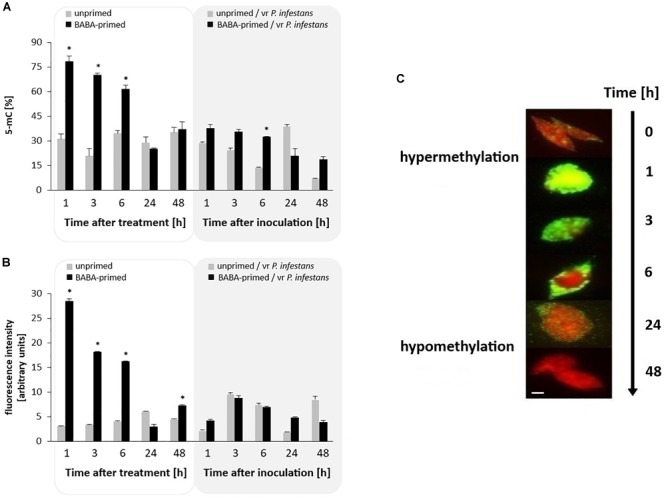
Global potato genome methylation in response to BABA treatment and challenge inoculation with *P. infestans* MP977 (at 72 hrs after BABA treatment). ELISA 5-mC assay **(A)**; Immunodetection of 5-mC estimated as Alexa Fluor^488^ fluorescence intensity **(B)**. Analyses were performed at 1–48 h after BABA (5 mM) treatment (white background) and 1–48 hpi (gray background) after challenge inoculation; the immunostaining pattern of representative nuclei at 0–48 h after BABA treatment **(C)**. The red color: DAPI staining (indicating lack of methylation); the green color: methylated DNA immunodetected with anti-5-mC antibody and Alexa Fluor^488^ goat anti-mouse IgG. The fluorescence signals for nuclei and 5-mC were pseudocolored red and green, respectively, using the ImageJ program. Scale bar = 5 μm. Light columns and dark columns refer to unprimed and primed plants, respectively. The negative control was provided by immunostaining without the secondary or primary antibody. In both cases, we observed no fluorescence of Alexa Fluor^488^ on the slide of leaf nuclei, which means that the antibodies were specific and showed no unspecific binding. Values represent mean ± SD of at least three independent experiments. Asterisks (^∗^) indicate values that differ significantly from unprimed (water treated) or unprimed and *P. infestans* inoculated potato leaves at α<0.05, respectively.

The 5-mC DNA ELISA assay revealed that the DNA methylation status of potato leaves increased upon BABA treatment since it was found to be 2-fold higher (from 1 to 6 h); then it diminished slightly in the following hours (Figure 4A). This indicates that during the first 6 h after BABA treatment the DNA methylation increased to 80% (hypermethylation state), followed by a decrease to 35% of 5-mC. Upon the challenge with *P. infestans* a loss in potato DNA methylation was noted both in primed and unprimed potato plants subjected to *P. infestans* inoculation.

### Nuclei Methylation Changes

The immunostaining patterns corresponding to 5-mC were investigated in the nuclei of potato leaf tissue. BABA pretreatment was associated with early and drastic hypermethylation (5-fold increase) from 1 to 6 h, followed by a rapid hypomethylation of DNA in potato leaf nuclei when compared to unprimed plants (Figure 4B,C and Supplementary Figure S2). The highest level of this modification, visible as green nuclei (corresponding to Alexa^488^ fluorescence), was observed at 1 h post BABA treatment; in contrast, the lowest level of 5-mC methylation was detected at 24 h since nuclei were visible red (corresponding to DAPI fluorescence). After pathogen ingress, the observed level of methylation in nuclei slightly differed in immunofluorescence intensity between the *P. infestans***-**challenged and BABA-primed inoculated leaf tissue at various time points after disease development (Figure 4B).

### Stress-Responsive Gene Expression and Induced DNA Methylation in Promoter

#### DNA Methylation Does Not Influence the Transcription of SA-Dependent Genes

**FIGURE 5:**
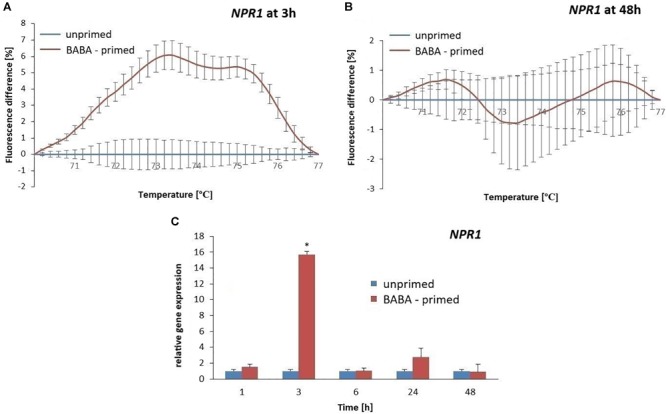
Gene expression and methylation level of *NPR1* promoter after BABA treatment in potato. Melting curves of the MS-HRM assay at 3 h **(A)** and 48 h after BABA treatment **(B)**. It is noted that higher fluorescence levels in BABA-treated plants compared to the unprimed showed enhanced DNA methylation of the *NPR1* promoter at 3 h after BABA treatment. The RT-qPCR analysis of *NPR1* expression in primed leaves of potato plants at 1–48 h after BABA treatment **(C)**. Values represent mean ± SD of at least three independent experiments. Asterisks (^∗^) indicate values that differ significantly from unprimed (water-treated) potato leaves at α<0.05, respectively.

**FIGURE 6:**
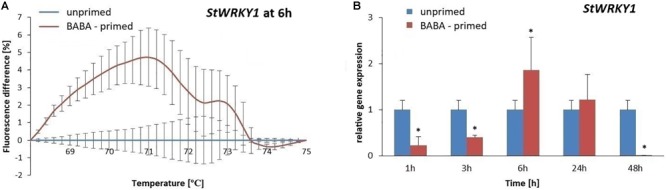
Gene expression and methylation level of *StWRKY1* promoter after BABA treatment in potato. Melting curves of the MS-HRM assay at 6 h after BABA treatment **(A)**. Details of the MS-HRM assay are given in the Materials and Methods. Higher fluorescence levels in BABA-treated plants compared to the unprimed showed enhanced DNA methylation of the *StWRKY1* promoter at 6 h. RT-qPCR analysis of *StWRKY1* expression in primed leaves of potato plants at 1–48 h after BABA treatment **(B)**. Values represent mean ± SD of at least three independent experiments. Asterisks (^∗^) indicate values that differ significantly from unprimed (water-treated) potato leaves at α<0.05, respectively.

**FIGURE 7:**
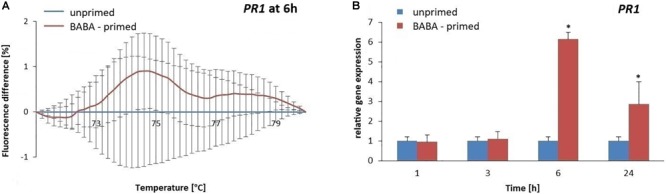
Gene expression and methylation level of *PR1* promoter after BABA treatment in potato. Melting curves of the MS-HRM assay at 6 h after BABA treatment **(A)**. Details of the MS-HRM assay are given in the Materials and Methods section. Fluorescence levels in BABA-treated plants compared to the unprimed showed no significant differences in the *PR1* promoter methylation upon BABA exposure. RT-qPCR analysis of *PR1* expression in primed leaves of potato plant at 1–48 h after BABA treatment **(B)**. Values represent mean ± SD of at least three independent experiments. Asterisks (^∗^) indicate values that differ significantly from unprimed (water treated) potato leaves at α<0.05, respectively.

To evaluate the methylation patterns in the promoter regions of selected SA-dependent defense genes, i.e., *NPR1, StWRKY1*, and *PR1*, upon the BABA-inducible DNA methylation we employed MS-HRM analyses. Potato exposure to BABA caused time-dependent changes in the methylation levels of the *NPR1* promoter. Initially, BABA induced a rapid increase in DNA methylation of the *NPR1* promoter at 3 h (5–6% rise in fluorescence intensity), followed by a decrease at 48 h, reaching similar levels as in the unprimed potato (Figure 5A,B and Supplementary Figures S3A,B). Unexpectedly, the high 5-mC level of the *NPR1* promoter at 3 h did not correlate with the high *NPR1* gene expression upon BABA treatment (Figure 5C). At the later time point (48 h) the induced 5-mC mark level at the *NPR1* promoter region reached the unprimed level since the transcript abundance of *NPR1* (6–48 h) was comparable to that in the unprimed potato. The *StWRKY1* promoter was partially methylated (6 h) based on the MS-HRM analysis and was not sufficiently effective in reducing *StWRKY1* transcription (Figure 6A,B and Supplementary Figure S3C). Our data showed an enhanced expression of *NPR1* and the *StWRKY1* transcription factor during the onset of the priming state that was accompanied by methylated patterns of the promoter regions of these pivotal genes for potato defense. MS-HRM analysis showed that the methylation state of the *PR1* promoter did not alter upon BABA exposure, whereas a higher accumulation of the *PR1* transcript (6 h) was found at 3 h (Figure 7A,B and Supplementary Figure S3D).

The obtained results revealed no association between promoter DNA methylation of SA-dependent genes and their expression levels in BABA-primed potato. This unexpected DNA methylation of the promoter regions results, reported in this study, might indicate that other epigenetic modifications may be associated with gene expression increase.

#### *R3a* Gene Promoter Loci-Specific Methylation Patterns

**FIGURE 8:**
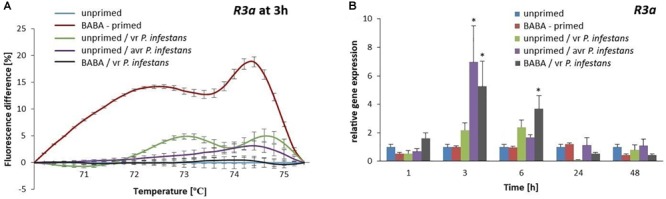
Gene expression and methylation level of *R3a* promoter after BABA treatment in potato. MS-HRM curves of the *R3a* promoter at 3 h after BABA or water treatment (unprimed) indicating its DNA methylated status, at 3 h after sequential treatment of BABA/vr *P. infestans* MP977 (primed-challenged), at 3 h after vr *P. infestans* MP977 inoculated (PTI-type of resistance) or Avr3a-encoding *P. infestans* MP946 inoculated (ETI-type of resistance) **(A)**, respectively. Values represent means of data ± SD of at least three independent experiments. RT-qPCR analysis of *R3a* gene expression in BABA-primed potato leaves followed by challenge inoculation with vr *P. infestans* MP977 or in unprimed potato only subjected to virulent *P. infestans* MP977 or Avr3a-encoding *P. infestans* MP946 inoculation **(B)**. Values represent mean ± SD of at least three independent experiments. Asterisks (^∗^) indicate values that differ significantly from those of unprimed (water treated) plants.

DNA methylation patterns of the *R3a* promoter became rapidly altered following BABA treatment and it was evident as divergent melting curve profiles were observed in the primed and unprimed potato (Supplementary Figure S4). The methylation level of the *R3a* promoter increased rapidly (15–20% rise in fluorescence intensity) immediately after the priming stimulus was recognized by the potato BABA receptor (6 h after BABA treatment) and such a promoter DNA methylation may have contributed to the observed downregulation of *R3a* expression (Figure 8A,B). In turn, after challenge inoculation with *P. infestans* MP977 the methylation level of the *R3a* promoter drastically diminished, since the melting curve profiles in the primed and unprimed potato plants were very similar (Supplementary Figure S4). In addition, the expression of *R3a* significantly increased in relation to the unprimed potato 3 h after *P. infestans* infection. The immediate upregulation (5-fold increase at 3 hpi) of *R3a*, playing a key role in the potato immune system, was a good indicator of potato in a primed state.

In order to better understand the putative correlation between the *R3a* gene expression and the methylated state of its promoter in relation to the type of immune response, we compared both ETI and PTI, defense responses, with BABA-primed ones (Figure 8A,B and Supplementary Figures S4A–D). Differences in DNA methylation patterns of the *R3a* promoter among the types of immune responses entail the speed and magnitude of *R3a* gene induction after inoculation. As early as 3 hpi after the challenge with the *Avr3a*-encoding *P. infestans* MP946 (ETI-type of defense), the highest (7-fold) increase in the *R3a* gene expression was observed with the lowest methylation level of its promoter (reflected by the percentage of fluorescence intensity shown in Figure 8A). A comparable, rapid and high (approx. 5-fold) increase in *R3a* gene expression with a low methylation level of its promoter was found in BABA-primed potato at 3 hpi after *P. infestans* MP977 inoculation. In turn, the unprimed potato inoculated with the virulent *P. infestans* MP977 (PTI-type of defense) showed only a 2-fold increase in gene transcription with a more methylated promoter sequence of *R3a* compared to the ETI and BABA-primed types of defense. These results indicate that a suitable DNA methylation level in the *R3a* gene promoter could be essential for its expression, which as a result may contribute to plant immunity in potato.

### DNA Methylation Level Is Correlated With Intergenerational Priming for Defense in Potato

**FIGURE 9:**
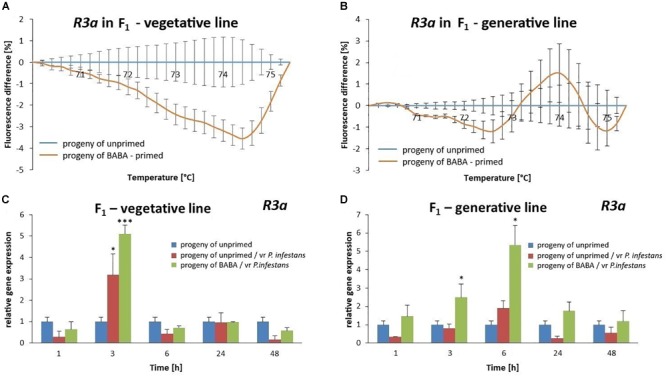
DNA methylation of the *R3a* promoter region involved in intergenerational priming for defense against vr *P. infestans* in potato. The MS-HRM melting curve of the *R3a* promoter region showed hypomethylation in the vegetative progeny of BABA-primed parents **(A)** and less methylated promoter of this gene in the generative progeny obtained from seeds **(B)**. Values represent means of data ± SD of at least three independent experiments. The RT-qPCR analysis of *R3a* gene expression after vr *P. infestans* inoculation in the offspring of BABA-primed plants obtained from tubers **(C)** or seeds **(D)**. Values represent mean ± SD of at least three independent experiments. Asterisks (^∗^) or (^∗∗∗^) indicate values that differ significantly from the progeny of unprimed (water-treated) potato leaves at α<0.05 and α<0.01, respectively, at each time point.

We analyzed whether changes in the DNA methylation patterns after BABA priming might provoke an intergenerational shift in the *R3a* gene expression. Indeed, the offspring of BABA-primed plants derived from tubers or seeds exhibited high levels of the *R3a* gene expression that correlated with an intergenerational resistance to vr *P. infestans* when compared with the unprimed potato (Figure 9C,D).

MS-HRM analysis showed that the *R3a* promoter region in the progeny potato derived from BABA-primed parents by propagation from tubers (F_1_) was highly hypomethylated, whereas progeny from seeds (F_1_) was only slightly hypomethylated when compared to potato plants from unprimed parents (Figure 9A,B and Supplementary Figure S5). Together, our data suggest that BABA-induced reversible changes in the methylation pattern of the *R3a* promoter that could contribute to improved functionality of R3a in parental plants, and probably also other *R* gene clusters in the tetraploid potato under biotic stress, and that these adaptive changes were passed onto their progeny.

## Discussion

It is generally accepted that primed plants show a faster and stronger activation of defense responses against pathogens after challenge inoculation. Much less is known on the coordination of epigenetic mechanisms associated with gene transcriptional reprogramming during the establishment of the primed state. Recent reports have underlined that DNA methylation guarantees stabilization and protects the genome against unwanted rearrangements, while targeted hypomethylation of specific loci is related with the acquisition of new traits or release of suppression of silent genes improving plant resistance to biotic factors (Zhu et al., 2015; López Sánchez et al., 2016; Viggiano and de Pinto, 2017; Tamiru et al., 2018).

S-adenosyl-L-homocysteine hydrolase plays a key role in the cellular AMC. Unlike human cells, in which impaired SAHH activity leads to severe diseases as a consequence of homocysteine over-accumulation (Tehlivets et al., 2013), in plant cells suppression of SAHH activity tends to increase resistance to various viruses (Yang et al., 2011; Cañizares et al., 2013). Previously we found that down-regulation of *SAHH* is linked to potato resistance against *P. infestans* (Arasimowicz-Jelonek et al., 2013). Similarly, other data showed that co-silencing of tomato *SAHH* enhances immunity to *Pseudomonas syringae* pv. *tomato* DC3000 (Li et al., 2015).

It was documented by Rocha et al. (2005) that mutations in the *AtSAHH1* gene caused a general reduction in the 5-mC level associated with the transcriptionally active chromatin structure in *Arabidopsis*. In transgenic tobacco, the expression of antisense RNA of *SAHH* resulted in DNA hypomethylation (Tanaka et al., 1997), while *Arabidopsis* supplied with a SAHH inhibitor (dihydroxypropyladenine) reduced the pool of methylated DNA (Baubec et al., 2010).

The results from the current study revealed that both *SAHH* (removing SAH) and *SAMS* (producing S-adenosylmethionine) were engaged in a modulation of the cellular methylating potential in BABA-primed potato. It is likely that BABA triggered epigenetic changes through modulation of the SAH/SAM ratio and initially potentiated the DNA methylation state in potato. In fact, drastic genome-wide DNA hypermethylation was observed shortly after BABA administration, as reflected by the 2-fold rise in global 5-mC detected by ELISA immunoassay (Figure 4A). The shift in DNA methylation was confirmed by immunostaining of primed potato leaf nuclei. In agreement with these observations, a rapid (3–6 h) up-regulation was noted for both *CMT3* and *DRM2*, engaged in *de novo* DNA methylation, accompanied by enhanced *ROS1* activation. Together, our results suggest that immediately after BABA treatment the primed potato reprogrammed the DNA methylation patterns toward hypermethylation. However, during the maintenance of the priming state, the excessive DNA methylation changed into demethylation with the active involvement of *StDML2* and probably also other DNA glycosylases.

Changes in *de novo* DNA methylation in a plant genome can be dynamic during the priming events. Moreover, defense priming has been divided into a priming phase, a post-challenged primed state and an inter- or transgenerational primed state (Balmer et al., 2015). Thus performing analyses of DNA methylation from the whole genome bisulfite sequencing data (WGBS) might lead to a misinterpretation of epigenetic responses related to the transcriptional state of genes while we are focusing on one time-point of the priming event.

Interestingly, the *Arabidopsis ros1* mutant showed enhanced RdDM and decreased plant resistance to bacteria (Yu et al., 2013). In addition, the triple *Arabidopsis* DNA demethylase mutant *rdd* (*ros1 dml2 dml3*) showed high susceptibility to *Fusarium oxysporum* with downregulation of many stress-responsive genes (Le et al., 2014).

Reports on DNA methylation and its relationship with plant resistance to pathogens seem to be of particular interest; so far, however, collected data are rather puzzling. It was found that three resistance inducers reduced the DNA methylation level providing resistance to powdery mildew in barley (Kraska and Schönbeck, 1993). In *Arabidopsis* virulent or avirulent strains of *Pseudomonas syringae* pv. *tomato* elicit the host plant DNA demethylation (Pavet et al., 2006). In contrast, *Arabidopsis* plants subjected to various types of biotic and abiotic stresses (i.e., salt, UVB, cold, heat, and flood) revealed a rise in global genome methylation correlated with stress adaptive changes that are transmitted to the progeny of stressed plants (Boyko et al., 2007, 2010; Bilichak et al., 2012). The analysis of total DNA methylation in two consecutive generations of TMV-infected tobacco showed a hypermethylated genome and an increased resistance to TMV, *Pseudomonas syringae* and *Phytophthora nicotianae* (Kathiria et al., 2010). However, in these findings, no clear correlation between DNA methylation and stress resistance was observed since the progeny of stressed tobacco or *Arabidopsis* were differentially methylated with the hypomethylated regions located in several LRR-containing loci or in euchromatic areas of nuclei (Boyko et al., 2010; Kathiria et al., 2010). Furthermore, genome-wide studies with various *Arabidopsis* mutants defective in CG (*met1-3*) or non-CG (*ddc, drm1-2, drm-2-2*, and *cmt3-11*) methylation showed enhanced resistance to *P. syringae* pv. *tomato* (Pst) DC3000 compared to the wild-type plant (Dowen et al., 2012). Also, Luna et al. (2012) documented that the hypomethylated DNA status in the *ddc Arabidopsis* mutant exhibited an intergenerational resistance phenotype to the bacteriumPstDC3000, but an enhanced susceptibility to the necrotrophic fungus *A. brassicicolla*.

Stress is known to induce the differential expression of various *R*-genes and defense-responsive genes, but the knowledge on the mechanism of *de novo* DNA methylation/active demethylation controlling their target recognition is still in its infancy (Ikeda and Kinoshita, 2009; Li et al., 2012; Bond and Baulcombe, 2015). Thus, we explored the impact of BABA treatment on the methylation state of the resistance gene *R3a* on potato, as well as the selected SA-dependent genes that might provide a background for long-term memory of host resistance to *P. infestans*. Time-dependent changes in the methylation status of the *NPR1* promoter upon BABA treatment were observed. At the onset of priming, the high 5-mC methylation state in the *NPR1* promoter negatively correlated with the cognate gene expression, but during the maintenance of the priming state, *NPR1* exhibited a reduction in promoter methylation and transcriptional down-regulation. How promoter methylation might activate *NPR1* gene expression remains to be investigated. One could speculate that DNA methylation may inhibit the binding of some transcription repressors or may promote the binding of some transcription activators (Wang et al., 2013).

Our results indicate that BABA treatment caused a 2-fold rise in *StWRKY1* expression at 6 h with no significant changes in the DNA methylation level of its promoter. According to Song et al. (2012), stress-responsive transcription factors may be influenced by both DNA methylation and histone modification in a region-specific manner, suggesting heterogeneity in the genome for gene expression regulated *via* epigenetic modifications under stress. Generally, the correlation between the DNA methylation status and gene expression is a highly multifaceted event, greatly dependent upon the type of methylation as well as the pattern of methylation within or outside the gene sequence (Niederhuth and Schmitz, 2017).

Our findings support a model where BABA can prime *PR1* gene expression without 5-mC alterations in its promoter region. This is in agreement with the study of Slaughter et al. (2012), where minimal changes in overall C-methylation in the *PR1* promoter region were observed both in the parent and descendants of BABA-primed *Arabidopsis*; however, *PR1* gene was highly responsive to priming and this effect was transferred to the next plant generation.

According to Dowen et al. (2012), inducible expression of some defense genes might not be directly caused by DNA methylation, but rather is the result of methylation-dependent alterations in the transcriptional network or hierarchic control of DNA methylation (Viggiano and de Pinto, 2017). Moreover, an important role devolves to cooperation between the stress-related gene expression and histone configuration, which may keep nucleosomes in the stand by state to facilitate rapid gene expression when needed (Alvarez-Venegas et al., 2007; Zhu et al., 2015). In support of the above, our previous data documented that both *NPR1* and *PR1*, early upon BABA treatment, showed a transient rise in H3K4me2, although later it was replaced by the H3K9me2 (or H3K27me3) mark that correlated with gene expression, and this effect was passed to the progeny resulting in improved protection against *P. infestans* (Meller et al., 2018).

In contrast to several published data on the methylation status of defense-responsive genes, there has been limited research on the regulation of *R*-gene expression by methylation. Precise regulation of the *R* gene expression is pivotal to prevent fitness costs and autoimmune responses in the absence of the pathogen. Although *R* genes are expressed constitutively at low levels, a rapid overexpression of the *R* genes contributes to improved resistance under biotic stress (de Vries et al., 2015). Generally, a high resistance level was associated with rapid *R* gene induction (Gu et al., 2005; Römer et al., 2007; Kramer et al., 2009); however, the expression of some *R* genes was occasionally very low (Tan et al., 2007), did not increase after infection (Huang et al., 2005) and it was influenced by various environmental factors or the host’s genetic background (Cao et al., 2007).

In the present study BABA alone did not activate *R3a* gene expression (Figure 8B), but triggered methylation of the *R3a* promoter region (Figure 8A). However, in successive hours after BABA treatment, an excessive DNA methylation status in potato was changed into demethylation. When the primed potato was challenged with vr *P. infestans* MP977, the promoter of *R3a* showed the demethylation state that correlated with its enhanced transcription. Considering the amplitude of *R3a* gene expression with the 5-mC level of the promoter, the obtained results showed the kinetics and speed of *R3a* gene induction in BABA-primed defense to be similar to the ETI–response rather than the PTI one. This finding might lead to the overall conclusion that DNA methylation is one of the factors controlling the kinetics of expression of both *R3a* and other defensive genes, while certain 5-mC-mediated changes might be involved in the intergenerational stress memory, leading to the formation of new epialleles conferring a broad-spectrum resistance against various pathogens.

We provided evidence that DNA methylation is a flexible mechanism that ultimately is able to modify the *R3a* resistance gene expression and initiate a favorable balance between silent and active gene transcription. BABA transiently activated rapid DNA methylation of the *R3a* promoter and triggered down-regulation of this gene in the absence of the pathogen. Furthermore, descendants of BABA-primed potato derived from tubers (mitotic intergenerational inheritance) or seeds (meiotic intergenerational inheritance) carrying a less methylated *R3a* promoter showed higher transcription of *R3a*, which correlated with enhanced intergenerational resistance to vr *P. infestans* MP977 when compared to the inoculated progeny of unprimed potato.

The obtained results confirmed once again that plants could integrate and “store” various signals into stress memory for future use or prepare their offspring for potential future assaults. To the best of our knowledge, this is the first report on the potato *R3a* gene activation/suppression by the stress-induced differentially methylated region of its promoter modifying the level of potato resistance to *P. infestans* both in the same and the successive generations. Our future efforts will focus on the recognition of the hierarchy of events that drives DNA methylation reprogramming in potato upon BABA treatment, such as ROS1 involvement, which seems to be preferentially engaged in counteracting *de novo* DNA methylation established by RdDM and probably plays a key role in stress-responsive gene expression associated with plant immunity.

## Author Contributions

JF-W and MA-J planned and designed the research. DK, BM, and AD performed the experiments, and collected and analyzed the data. AB-Z performed the immunostaining procedure. JF-W wrote the manuscript.

## Conflict of Interest Statement

The authors declare that the research was conducted in the absence of any commercial or financial relationships that could be construed as a potential conflict of interest.

## Footnotes

1 imagej.nih.gov

## Supplementary Material

FIGURE S1 Schematic representation of the experimental design. The parental line of “Sarpo Mira” potato genotype (F_0_) was treated once with 5 mM BABA (B_0_). The progeny (F_1_) grown from tubers or seeds were not again treated with BABA, but only inoculated with vr *P. infestans*- at the stage of 10 compound leaves. Abbreviations: the parental F_0_ line control plants – C_0_ and BABA-primed – B_0_, the offspring of control plants – C_V_ and C_G_, the primed plants of the vegetative F_1_ line – B_V_ and the generative F_1_ line – B_G_.

FIGURE S2 Global potato nuclei methylation study in response to BABA exposure. A representative image gallery of approx. 168 nuclei at 1, 6 and 48 h upon BABA treatment, respectively. The red color: DAPI staining (lack of methylation); the green color: methylated DNA was immunodetected with anti-5-methylcytosine (5-mC) antibody and Alexa Fluor^488^ goat anti-mouse IgG. The red and green colors were added using ImageJ software. Scale bar = 5 μm.

FIGURE S3 Methylation levels of *NPR1, StWRKY1*, and *PR1* promoter after BABA treatment in primed potato. MS-HRM curves of the DNA methylated state of the *NPR1* promoter at 3 and 48 h **(A,B)**; the *StWRKY1* promoter at 6 h **(C)**; the *PR1* promoter at 6 h after BABA treatment in primed potato **(D)**. Fluorescence values of BABA-treated plants (red line) were compared to the unprimed ones (blue line) as shown in the figure. Values represent mean ± SD of at least three independent experiments.

FIGURE S4 Methylation levels of *R3a* promoter in potato. MS-HRM curves of the DNA methylated state of the *R3a* promoter at 3 h after BABA treatment **(A)**; 3 h after vr *P. infestans* MP977 inoculation **(B)**; 3 h after vr *P. infestans* PM977 inoculation **(C)**; or after sequential treatment of BABA/vr *P. infestans* MP977 **(D)**, respectively. Fluorescence values of samples were compared to the control as shown in the figure. Values represent mean ± SD of at least three independent experiments.

FIGURE S5 Methylation levels of *R3a* promoter in intergenerational potato. Melting curves of the MS-HRM promoter region of *R3a* before vr *P. infestans* MP977 inoculation in the vegetative progeny of BABA-primed parents **(A)**, and the generative progeny obtained from seeds **(B)**. Fluorescence values of samples were compared to the control (progeny of unprimed plants) as shown in the figure. Values represent mean ± SD of at least three independent experiments.

TABLE S1 Numerical data of melting curves obtained from MS-HRM analysis for the tested genes.

TABLE S2 Primers used for RT-qPCR and MS-HRM analyses.
