# Supplementary figures and images for: BABA-Induced DNA Methylome Adjustment to Intergenerational Defense Priming in Potato to Phytophthora infestans
Source: Front Plant Sci. 2019 May 31;10:650. doi: 10.3389/fpls.2019.00650 (PMC6554679; doi:10.3389/fpls.2019.00650)

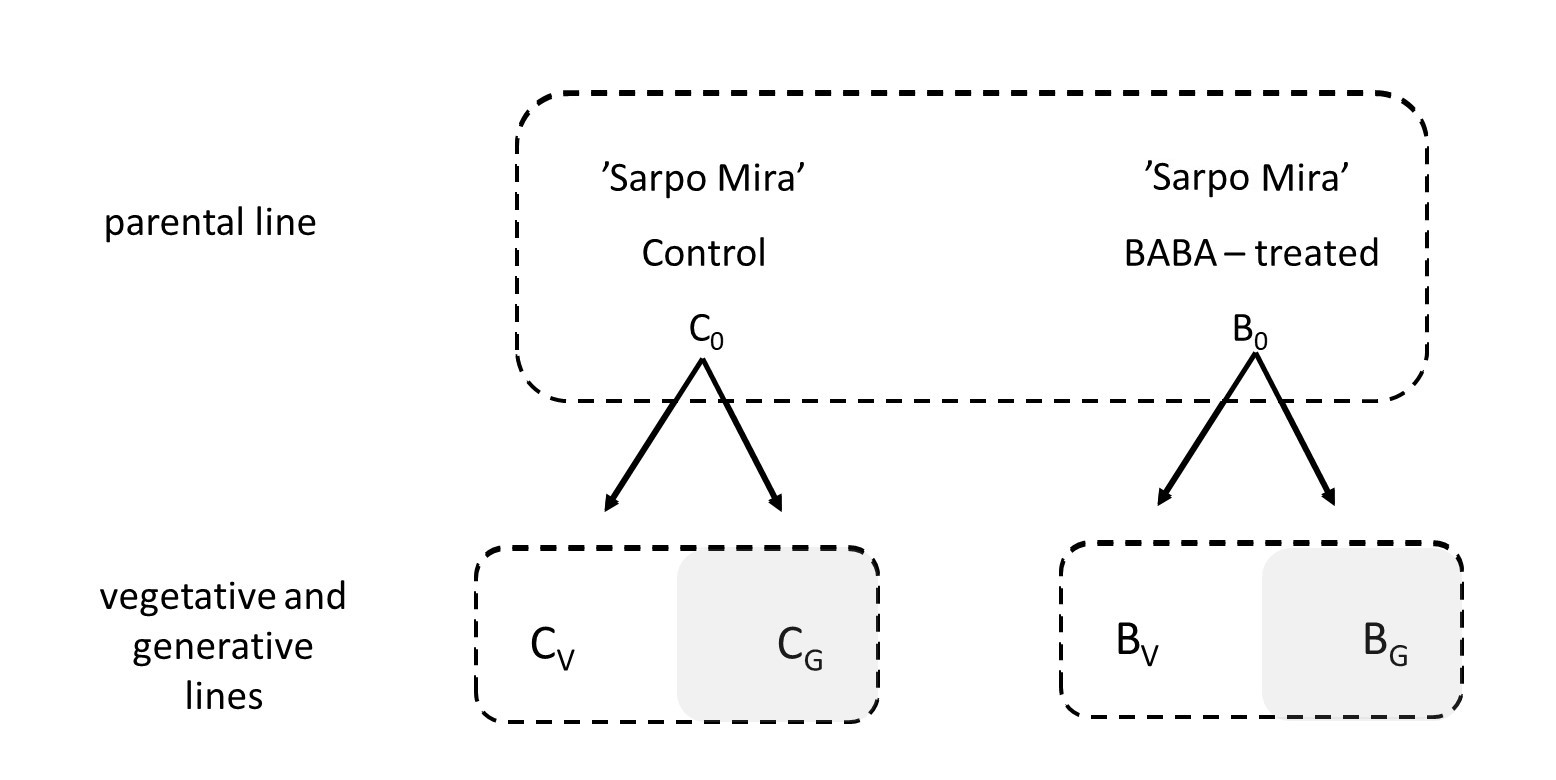

Supplement: FIGURE S1 — Schematic representation of the experimental design. The parental line of “Sarpo Mira” potato genotype (F0) was treated once with 5 mM BABA (B0). The progeny (F1) grown from tubers or seeds were not again treated with BABA, but only inoculated with vr P. infestans- at the stage of 10 compound leaves. Abbreviations: the parental F0 line control plants – C0 and BABA-primed – B0, the offspring of control plants – CV and CG, the primed plants of the vegetative F1 line – BV and the generative F1 line – BG. [file Image_1.JPEG]

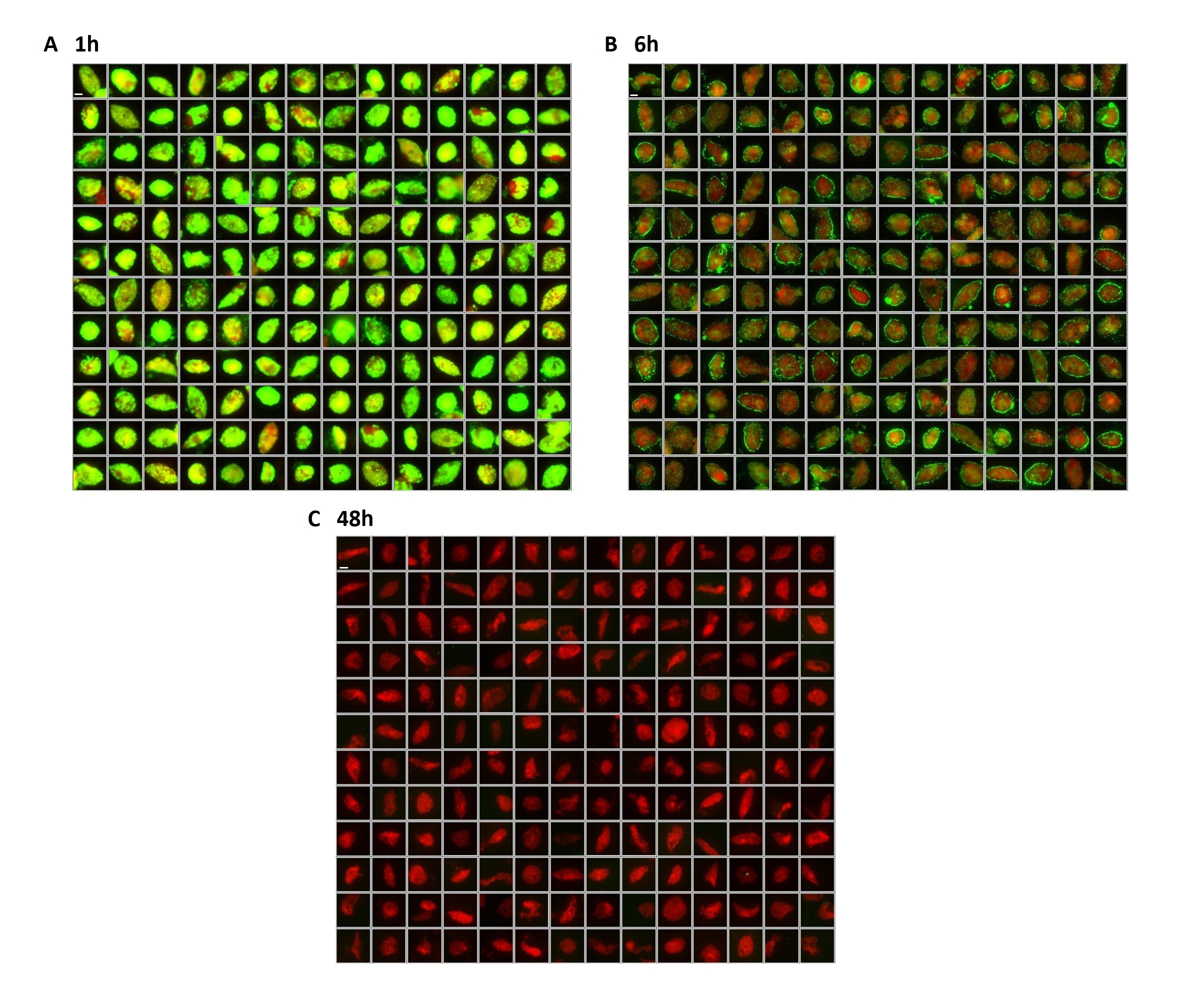

Supplement: FIGURE S2 — Global potato nuclei methylation study in response to BABA exposure. A representative image gallery of approx. 168 nuclei at 1, 6 and 48 h upon BABA treatment, respectively. The red color: DAPI staining (lack of methylation); the green color: methylated DNA was immunodetected with anti-5-methylcytosine (5-mC) antibody and Alexa Fluor488 goat anti-mouse IgG. The red and green colors were added using ImageJ software. Scale bar = 5 μm. [file Image_2.JPEG]

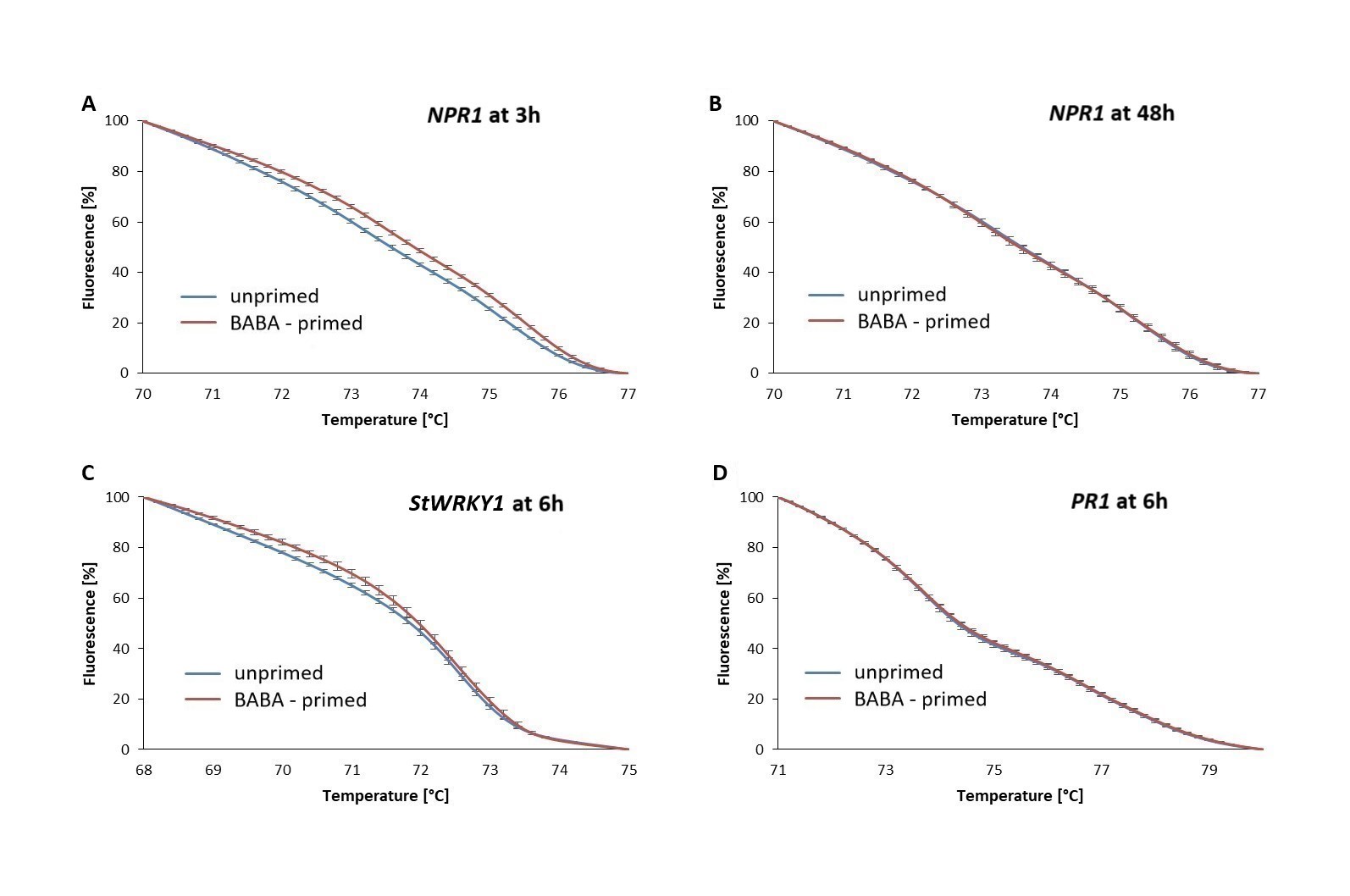

Supplement: FIGURE S3 — Methylation levels of NPR1, StWRKY1, and PR1 promoter after BABA treatment in primed potato. MS-HRM curves of the DNA methylated state of the NPR1 promoter at 3 and 48 h (A,B); the StWRKY1 promoter at 6 h (C); the PR1 promoter at 6 h after BABA treatment in primed potato (D). Fluorescence values of BABA-treated plants (red line) were compared to the unprimed ones (blue line) as shown in the figure. Values represent mean ± SD of at least three independent experiments. [file Image_3.JPEG]

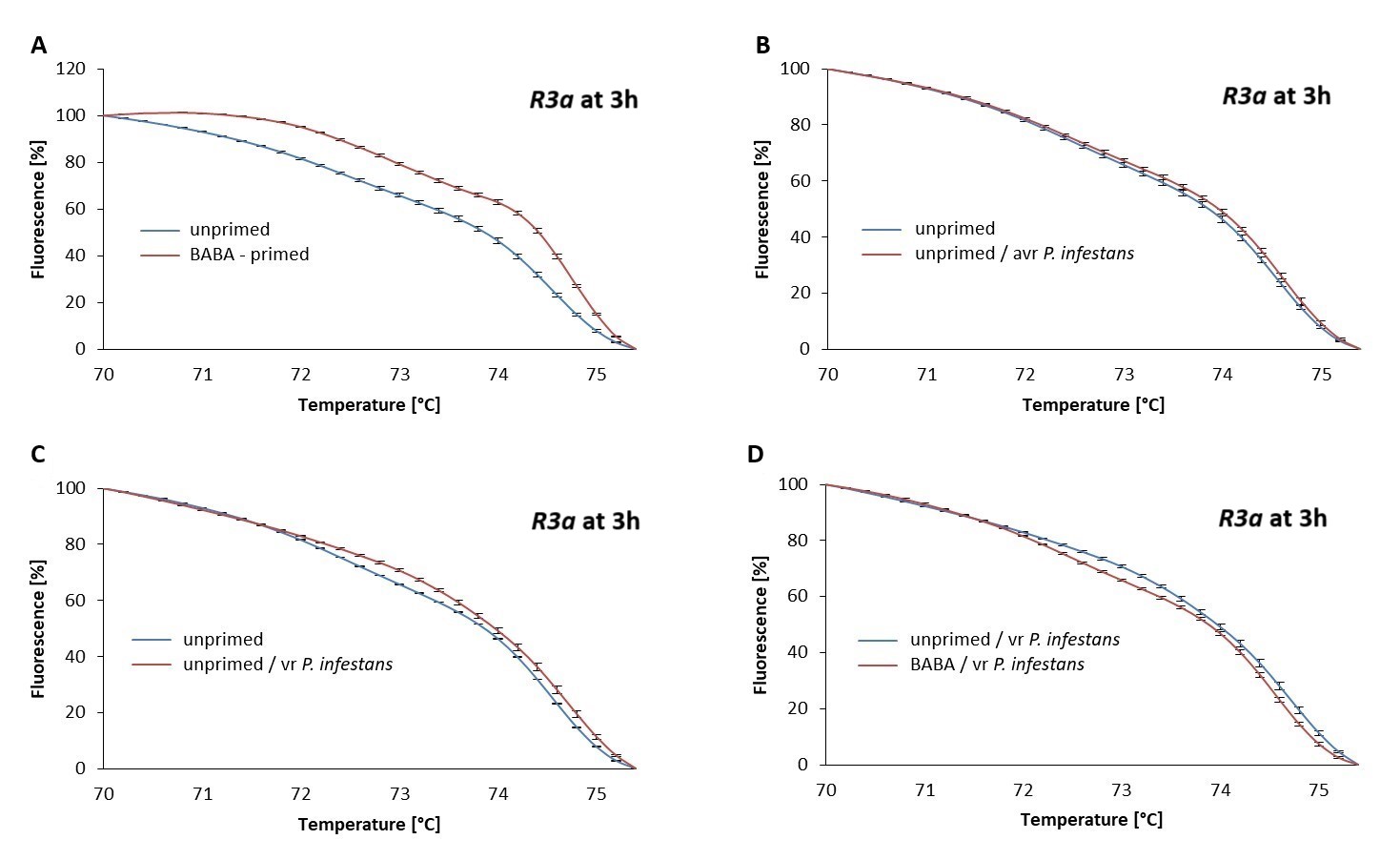

Supplement: FIGURE S4 — Methylation levels of R3a promoter in potato. MS-HRM curves of the DNA methylated state of the R3a promoter at 3 h after BABA treatment (A); 3 h after vr P. infestans MP977 inoculation (B); 3 h after vr P. infestans PM977 inoculation (C); or after sequential treatment of BABA/vr P. infestans MP977 (D), respectively. Fluorescence values of samples were compared to the control as shown in the figure. Values represent mean ± SD of at least three independent experiments. [file Image_4.JPEG]

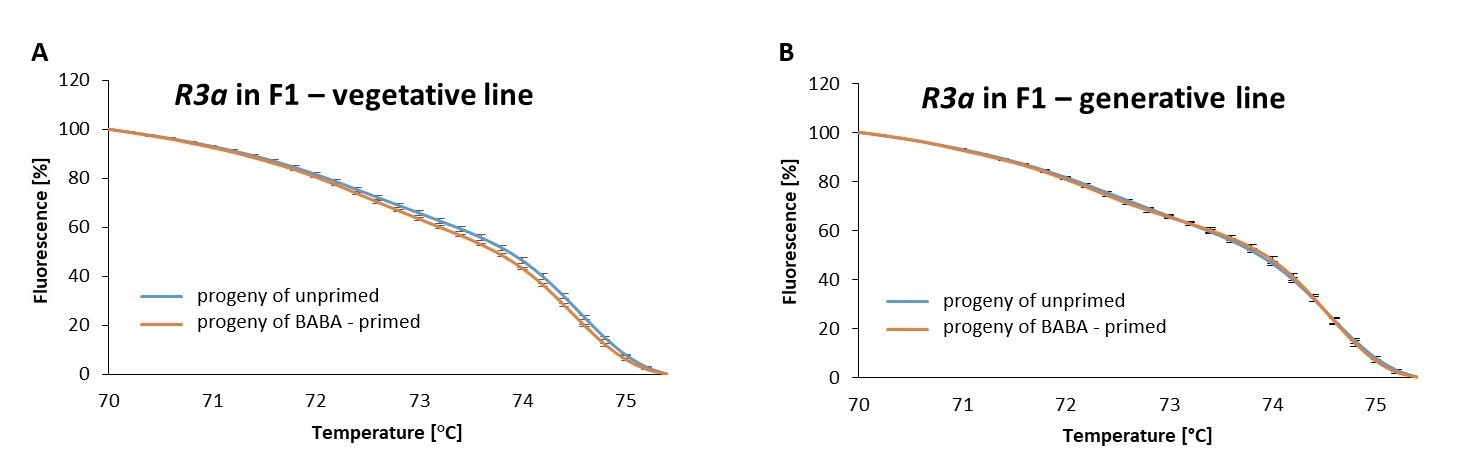

Supplement: FIGURE S5 — Methylation levels of R3a promoter in intergenerational potato. Melting curves of the MS-HRM promoter region of R3a before vr P. infestans MP977 inoculation in the vegetative progeny of BABA-primed parents (A), and the generative progeny obtained from seeds (B). Fluorescence values of samples were compared to the control (progeny of unprimed plants) as shown in the figure. Values represent mean ± SD of at least three independent experiments. [file Image_5.JPEG]
