# Supplementary material for: BABA-Induced DNA Methylome Adjustment to Intergenerational Defense Priming in Potato to Phytophthora infestans
Source: Front Plant Sci. 2019 May 31;10:650. doi: 10.3389/fpls.2019.00650 (PMC6554679; doi:10.3389/fpls.2019.00650)
Supplement: TABLE S2 — Primers used for RT-qPCR and MS-HRM analyses. [file Table_2.DOCX]

| Primer | Sequence | Accession | Melting temperature [°C] | Product length [bp] |
| --- | --- | --- | --- | --- |
| *ef1α* F | ATTGGAAACGGATATGCTCCA | AB061263 | 60,4 | 93 |
| *ef1α* R | TCCTTAACCTGAACGCCTGTCA |  | 59,6 |  |
| *18s rRNA* F | GGGCATTCGTATTTCATAGTCAGAG | X67238 | 59,6 | 101 |
| *18s rRNA* R | CGGTTCTTGATTAATGAAAACATCCT |  | 58,5 |  |
| *Pitef1* F | ATGACTCGCCTCGGTGATTA | XM_002904671.1 | 61,8 | 106 |
| *Pitef1* R | TCCACACACACAAAGTGCATCA |  | 59,7 |  |
| *SAMS* F | TCAACCCATCAGGTCGCTTC | NM_001318549.1 | 60,8 | 127 |
| *SAMS* R | AGGCAACCACACTCTTTGCT |  | 58,8 |  |
| *SAHH* F | CATGGCTTCCCGTACTGAAT | DQ252503.1 | 58,8 | 86 |
| *SAHH* R | TCACCTTTCCAGGCAAAAAC |  | 57,7 |  |
| *MET1* F | CGGAGCGTATGGAGTTCCTC | NM_001247819.2 | 60,0 | 90 |
| *MET1* R | ATTGGTTCTGGCCACTCTGG |  | 60,0 |  |
| *CMT3* F | TTGTGACGAGAGCTGAACCC | XM_004252792.3 | 60,0 | 97 |
| *CMT3* R | GGAAAACCTTGGAGCCTTGC |  | 60,0 |  |
| *DRM2* F | AAGTTGGGGTTCAGTGTTTGC | XM_015312485.1 | 59,5 | 95 |
| *DRM2* R | TGTGCCACAGCAAAAGCATA |  | 58,7 |  |
| *StDML2* F | CAGTGAGCCATTCCCCGATT | XM_006344952.2 | 60,1 | 87 |
| *StDML2*R | GGGAAACCATGGAGAGCCAA |  | 60,0 |  |
| *ROS1* F | GGGGTTTTTGGTTCCTCAAT | XM_015314854 | 56,7 | 91 |
| *ROS1* R | TCAAGCCCATGCTATTACCC |  | 58,8 |  |
| *NPR1* F | GGTGCACCGATGCATTTTGT | XM_006357647.2 | 59,8 | 145 |
| *NPR1* R | AATAGGCGAGCACACTGACC |  | 60,8 |  |
| *StWRKY1* F | TCAGCATCATCGTCGTCATC | AJ278507.1 | 58,8 | 134 |
| *StWRKY1* R | TGCCATTAGTCCCAAGAACC |  | 58,8 |  |
| *PR1* F | GGGAGAAGCCAAACTACAACTATG | AJ250136.1 | 55,7 | 108 |
| *PR1* R | ACGAGCCCGACCACAACC |  | 54,9 |  |
| *R3a* F | GGAACAGCCAACTGGTGAGA | AY849382.1 | 53,8 | 107 |
| *R3a* R | GTGCCAATCCTGTACCCACA |  | 53,8 |  |
| HRM*NPR1* F | AAGAAGATAGTTGATGTATAGAGGATAA | XM_006357647.2 | 64,8 | 183 |
| HRM *NPR1* R | CAATACACAAATCAAAATAAAAAAA |  | 64,8 |  |
| HRM *StWRKY1* F | GTGTGGAGTTATTTTTTTATTAGGTTATTT | AJ278507.1 | 58,2 | 248 |
| HRM *StWRKY1* R | ACTCATTAAACACCCCAAACTTTAC |  | 58,9 |  |
| HRM *PR1* F | ATTAGATTTAAAGTAAAATGGGGTTGTT | AJ250136.1 | 59,1 | 240 |
| HRM *PR1* R | CCTTAACAAAATTCTCCCCTACAC |  | 59,4 |  |
| HRM *R3a* F | TGTTTTATTTGATGGGAAAGGTATAA | AY849382.1 | 59,1 | 202 |
| HRM *R3a* R | AATCAACAAATAATTTAAACAACATTC |  | 59,3 |  |
